# Simulation and Parameter Optimization of Inserting–Extracting–Transporting Process of a Seedling Picking End Effector Using Two Fingers and Four Needles Based on EDEM-MFBD

**DOI:** 10.3390/plants15020291

**Published:** 2026-01-18

**Authors:** Jiawei Shi, Jianping Hu, Wei Liu, Mengjiao Yao, Jinhao Zhou, Pengcheng Zhang

**Affiliations:** Jiangsu Provincial Key Laboratory of Hi-Tech Research for Intelligent Agricultural Equipment, Jiangsu University, Zhenjiang 212013, China; 2112116016@stmail.ujs.edu.cn (J.S.); mario_liu@ujs.edu.cn (W.L.); 2112216018@stmail.ujs.edu.cn (M.Y.); 2112316042@stmail.ujs.edu.cn (J.Z.); 2112416036@stmail.ujs.edu.cn (P.Z.)

**Keywords:** automatic transplanter, seedling picki/ng end effector, EDEM-MFBD, Box–Behnken test, parameter optimization

## Abstract

This paper aims to address the problem of the low success rate of seedling picking and throwing, and the high damage rate of pot seedling, caused by the unclear interaction and parameter mismatch between the seedling picking end effector and the pot seedling during the seedling picking and throwing process of automatic transplanters. An EDEM–RecurDyn coupled simulation was conducted, through which the disturbance of substrate particles in the bowl body during the inserting, extracting, and transporting processes by the seedling picking end effector was visualized and analyzed. The force and motion responses of the particles during their interaction with the seedling picking end effector were explored, and the working parameters of the seedling picking end effector were optimized. A seedling picking end effector using two fingers and four needles is taken as the research object, a kinematic mathematical model of the seedling picking end effector is established, and the dimensional parameters of each component of the end effector are determined. Physical characteristic tests are conducted on Shanghai bok choy pot seedlings to obtain relevant parameters. A discrete element model of the pot seedling is established in EDEM 2022 software, and a virtual prototype model of the seedling picking end effector is established in Recurdyn 2024 software. Through EDEM-Recurdyn coupled simulation, the force and movement of the substrate particles in the bowl body during the inserting, extracting, and transporting processes of the seedling picking end effector under different operating parameters were explored, providing a theoretical basis for optimizing the working parameters of the end effector. The inserting and extracting velocity, transporting velocity, and inserting depth of the seedling picking end effector were used as experimental factors, and the success rate of seedling picking and throwing, and the loss rate of substrate, were used as evaluation indicators; single-factor tests and three-factor, three-level Box–Behnken bench tests were conducted. Variance analysis, response surface methodology, and multi-objective optimization were performed using Design-Expert 13 software to obtain the optimal parameter combination: when the inserting and extracting velocity was 228 mm/s, the transporting velocity was 264 mm/s, the inserting depth was 37 mm, the success rate of seedling picking and throwing was 97.48%, and the loss rate of substrate was 2.12%. A verification experiment was conducted on the bench, and the success rate of seedling picking and throwing was 97.35%, and the loss rate of substrate was 2.34%, which was largely consistent with the optimized results, thereby confirming the rationality of the established model and optimized parameters. Field trial showed the success rate of seedling picking and throwing was 97.04%, and the loss rate of substrate was 2.41%. The error between the success rate of seedling picking and throwing and the optimized result was 0.45%, indicating that the seedling picking end effector has strong anti-interference ability, and verifying the feasibility and practicality of the established model and optimized parameters.

## 1. Introduction

China’s vegetable planting area has exceeded 22 million hectares, with an annual output exceeding 830 million tons, essentially achieving a stable supply throughout the year [1,2]. However, the low level of mechanization in vegetable planting has become a key bottleneck restricting the high-quality development of the vegetable industry, and it urgently needs to be addressed and improved [3,4,5,6,7,8]. Seedling transplanting is the main method of vegetable planting. Due to increasing demand for transplanting, automatic transplanters are considered the main development trend of mechanized transplanting. The seedling-picking end effector is the core component of the automatic transplanter, and its structural design and working parameters have a significant impact on the transplanting performance of the entire machine [9,10,11,12,13,14,15].

To solve the technical difficulties in the seedling picking process, scholars at home and abroad have conducted a lot of research. ISLAM et al. [16] designed a needle-type seedling picking end effector based on a cam–linkage compound transmission mechanism, in which the opening–closing motion and downward inserting of the picking needles are realized through coordinated cam and linkage actions. CHOI et al. [17] proposed a clamping-needle-type seedling picking end effector, in which the reciprocating motion of a piston rod drives the opening and closing of the clamping needles, thereby accomplishing the seedling picking and throwing process. Zhang et al. [18] developed a four-finger opening–closing seedling picking end effector, where a pneumatic cylinder drives an intermediate linkage to achieve synchronous opening and closing of the picking fingers. Ji et al. [19] proposed a two-needle seedling picking end effector, in which the inclination angle of the clamping needles gradually increases during the inserting process, thereby enhancing the clamping force on the bowl body. The above studies all determined the optimal parameters of the seedling end effector through multi-factor orthogonal experiments; this type of method regards the bowl body as a continuous body, and lacks analysis of the mechanical interaction between the seedling end effector and the substrate particles in the bowl body through simulation of discrete element method (EDEM) and multiple flexible body dynamics (MFBD). Zhang et al. [20] established a discrete element model of the bowl body using EDEM to analyze the seedling pushing and picking processes of a push-out clamping-type seedling picking end effector, and optimized both structural and operating parameters. Du et al. [21] conducted single-factor and orthogonal experimental studies on the inserting and extracting processes of a plug-tray seedling picking end effector based on EDEM simulations. Cui et al. [22] employed EDEM–RecurDyn co-simulation to investigate the mechanical characteristics of a seedling picking end effector based on a cylindrical cam during the inserting process into the bowl body. The above studies used EDEM-RecurDyn co-simulation to explore the interaction relationship between the seedling picking end effector and the substrate particles in the bowl body to some extent. However, they mainly focus on the inserting and extracting stages, generally neglecting the transporting stage after the pot seedling is extracted. In fact, during the transporting stage, the pot seedling needs to undergo spatial displacement and attitude changes with the end effector while being held in place. During this process, the substrate particles are continuously subjected to inertial forces, clamping forces, and gravity, which can easily lead to the loosening, fragmentation, or even complete destruction of the bowl body. Analyzing only the inserting or extracting stages is insufficient to fully reflect the force characteristics and operational reliability of the seedling picking end effector under actual working conditions, and it also fails to provide a complete and accurate theoretical basis for the systematic optimization of operational parameters. Therefore, it is urgent to consider inserting, extracting, and transporting as a continuous seedling picking process and systematically study the dynamic mechanical interaction mechanism between the seedling picking end effector and the substrate particles in the bowl body.

**Figure 1:**
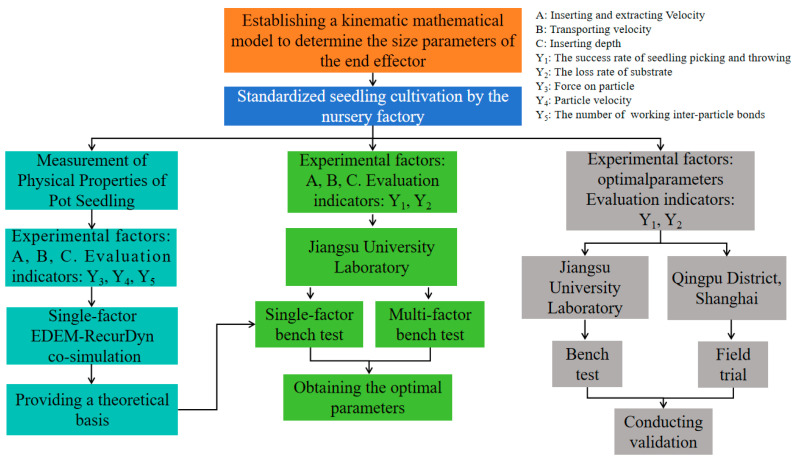
Research scheme.

In summary, based on the whole row reciprocating seedling picking mechanism, a seedling picking end effector using two fingers and four needles is taken as the research object, a kinematic mathematical model of the seedling picking end effector is established, and the dimensional parameters of each component of the end effector are determined. Based on EDEM–RecurDyn co-simulation, the inserting, extracting, and transporting processes are incorporated into a unified analysis framework, and the force and motion behaviors of substrate particles in the bowl body during the inserting, extracting, and transporting of the seedling picking end effector under different operating parameter conditions are systematically investigated, providing a theoretical basis for the optimization of operating parameters of the seedling picking end effector. Single-factor and three-factor three-level Box–Behnken tests were conducted on the seedling-picking end effector to determine the optimal working parameters. Bench testing and field trial of the seedling picking end effector were also carried out to verify the rationality and feasibility of the optimal parameter combination design, providing a theoretical basis and reference for optimizing the working parameters of the seedling-picking end effector of the automatic transplanter. (as shown in Figure 1).

## 2. Materials and Methods

### 2.1. The Structural Composition of the Seedling Picking Mechanism and End Effector

**Figure 2:**
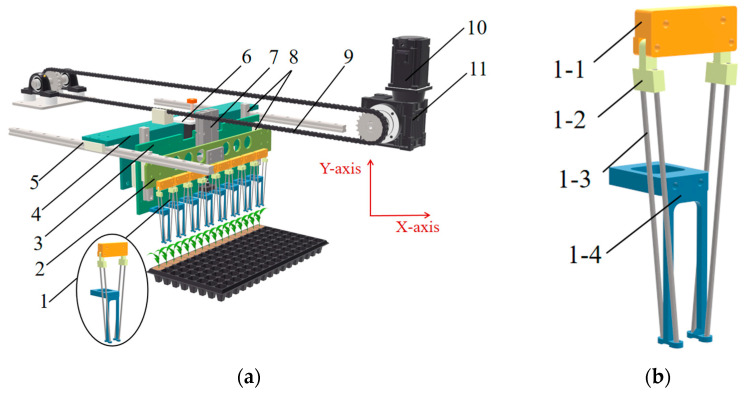
(**a**) Schematic diagram of the seedling picking mechanism: 1. End effector; 2. Clamping needle moving plate; 3. End effector moving plate in the Y-axis direction; 4. End effector moving plate in the X-axis direction; 5. Guide rail slider mechanism in the X-axis direction; 6. Lead screw motor; 7. Lifting cylinder; 8. Guide rail slider mechanism in the Y-axis direction; 9. Chain; 10. Servo motor; 11. Reducer. (**b**) Schematic diagram of the seedling picking end effector 1-1. Clamping needle fixing plate; 1-2. Clamping needle connecting block; 1-3. Clamping needle; 1-4. Seedling pushing ring.

The seedling picking mechanism mainly consists of 8 end effectors, a clamping needle moving plate, an end effector moving plate in the X-axis direction, an end effector moving plate in the Y-axis direction, a guide rail slider mechanism in the X and Y axes, a lead screw motor, a lifting cylinder, a chain, a servo motor, and a reducer, as shown in Figure 2a. The end effector mainly consists of a clamping needle fixing plate, 4 clamping needle connecting blocks, 4 clamping needles, and a seedling pushing ring, as shown in Figure 2b.

### 2.2. The Working Principle of the Seedling Picking End Effector

**Figure 3:**
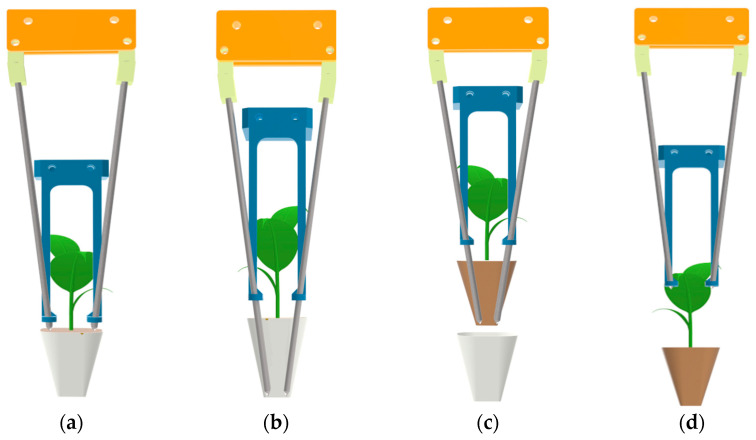
Schematic diagram of the working principle of the seedling picking effector. (**a**) The end effector reaches the seedling picking position. (**b**) The end effector inserts into the pot seedling. (**c**) The end effector extracts the pot seedling. (**d**) The end effector throws the pot seedling.

At the beginning of the seedling picking movement, the servo motor drives the end effector moving plate in the X-axis direction via a chain to move along the X-axis direction, enabling the end effector moving to the seedling picking position in the X-axis direction. The screw motor drives the end effector moving plate in the Y-axis direction to move along the negative Y-axis direction, enabling the end effector to move to the seedling picking position in the Y-axis direction, as shown in Figure 3a. After reaching the seedling picking position, the lifting cylinder extends the piston rod, which drives the clamping needle moving plate to move along the negative Y-axis direction, enabling the clamping needle to insert into the bowl body at a set depth, as shown in Figure 3b. After the end effector completes the inserting action, the lead screw motor drives the end effector moving plate in the Y-axis direction to move along the positive Y-axis, enabling the end effector to extract the pot seedling out of the hole in the seedling tray, as shown in Figure 3c. After the end effector completes the extracting action, the servo motor drives the end effector moving plate in the X-axis direction via the chain to move along the negative X-axis direction, enabling the end effector to move to the seedling throwing position in the X-axis direction. The lifting cylinder retracts the piston rod, which drives the clamping needle moving plate to move along the positive Y-axis direction, enabling the clamping needle to retract. The seedling pushing ring pushes the pot seedling away from the end effector, completing the seedling throwing action, as shown in Figure 3d.

### 2.3. The Design of Structural Parameters of the Seedling Picking End Effector

**Figure 4:**
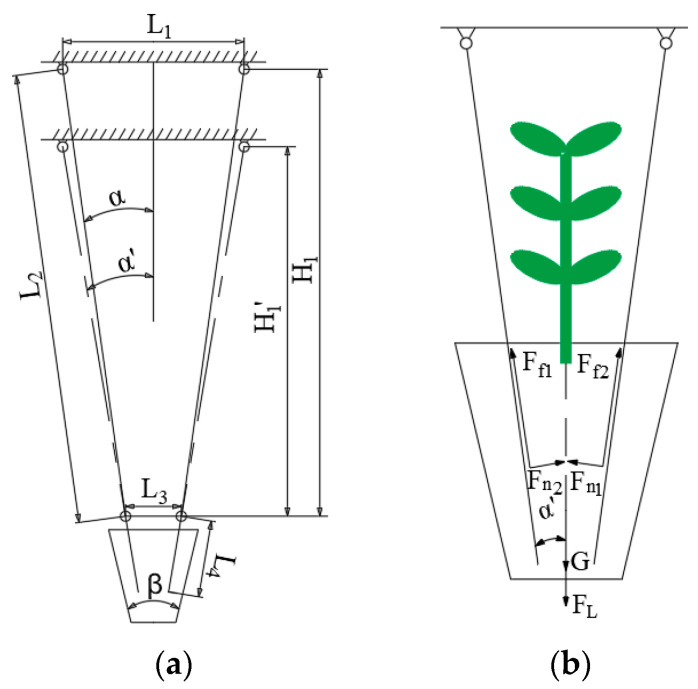
Schematic diagram of the kinematic and mechanical models of the end effector. (**a**) The kinematic model of the end effector. (**b**) The mechanical model of the end effector.

The structural parameters of the seedling picking end effector must be designed based on the characteristics of the target object, including the size of the hole, and the mechanical properties of the interaction between the clamping needle and the bowl body. A schematic diagram of the kinematic model of the seedling picking end effector is constructed based on its composition and working principle, as shown in Figure 4a. *L*_1_ is the installation distance between the tips of the two clamping needles; *L*_2_ is the length of the clamping needle; *L*_3_ is the distance between the two through holes of the seedling pushing ring; *L*_4_ is the distance of the clamping needle extending out of the through hole of the seedling pushing ring when it fully clamps the bowl body; *H*_1_ is the vertical distance between the tip of the clamping needle and the through hole of the seedling pushing ring when the clamping needle is in its initial state; *H*_1′_ is the vertical distance between the tip of the clamping needle and the through hole of the seedling pushing ring when the clamping needle is clamped; *α* is the angle between the clamping needle and the vertical direction when the clamping needle is in its initial state; *α*’ is the angle between the clamping needle and the vertical direction when the clamping needle is clamped; and *β* is the angle between the two walls of the hole.

The geometric relationships of the moving parts of the seedling picking end effector are shown in the following formula:

(1) $$\left\{\begin{array}{c} {2L}_{2}sin\alpha +L_{3}=L_{1}\\ 2(L_{2}-L_{4})sin\alpha^{\prime }+L_{3}=L_{1}\\ tan\alpha =\frac{L_{1}-L_{3}}{2H_{1}}\\ tan\alpha^{\prime }=\frac{L_{1}-L_{3}}{2H_{1^{\prime }}} \end{array}\right.$$

The seedling picking effector operates on a 128-hole seedling tray. The dimension of the upper surface of each hole is 32 mm × 32 mm, the dimension of the lower surface is 13 mm × 13 mm, and the depth is 42 mm. The angle *β* between the two walls is 22°. To ensure successful seedling picking, the distance *L*_3_ between the two through holes of the seedling pusher ring should be less than 32 mm. Considering the overall size of the seedling picking mechanism and the hole size, *L*_1_ is set to 52 mm and *L*_3_ to 21 mm. Based on previous research by the research group [23], the diameter of the clamping needle is set to 3 mm, and the initial angle of the clamping needle should not be greater than the angle between the two walls of the hole; therefore, the angle *α* is set to 7°. The angle when clamping the pot should be greater than or equal to the angle between the two walls of the hole, and therefore, the angle α’ is set to 11°. Based on the above parameter relationships, the geometric parameters of the end effector were finally determined, as shown in Table 1.

A force analysis was performed on the end effector during seedling picking, as shown in Figure 4b. When the total extracting force *F*_C_ is greater than the total resistance *F*_T_, the pot seedling can be successfully extracted, as shown in the following formula:

(2) $$\left\{\begin{array}{c} 2\left(F_{n1}+F_{n2}\right)\cdot sin\alpha^{\prime }+2\left(F_{f1}+F_{f2}\right)\times cos\alpha^{\prime }=F_{C}\\ F_{f}=\mu \cdot F_{n}\\ F_{T}=G+F_{L}\\ F_{C}>F_{T} \end{array}\right.$$

where *F*_n1_ and *F*_n2_ are the normal forces exerted by the two clamping needles on the bowl body, N; *F*_f1_ and *F*_f2_ are the frictional forces generated between the clamping needles and the bowl body when clamped, N; *α*’ is the angle between the clamping needles and the vertical direction when clamped, 11°; *μ* is the coefficient of friction between the clamping needles and the bowl body, 0.4; *F*_C_ is the total extracting force, N; *F*_T_ is the total resistance, N; G is the weight of the pot seedling, N; and *F*_L_ is the adhesive force between the bowl body and the hole, N. Assuming the bowl body structure is uniform, then the forces between the clamping needles and the bowl body are *F*_n1_ = *F*_n2_ = *F*_n_, *F*_f1_ = *F*_f2_ = *F*_f_.

## 3. Coupled Simulation Based on EDEM-Recurdyn

During the actual seedling and throwing process of the end effector, the substrate in the bowl body is in a complex interactive environment composed of particle mechanics and multibody dynamics [24,25]. It is necessary to analyze the influence of different working parameters on the substrate particles of the pot through EDEM-RecurDyn coupled simulation.

### 3.1. Establishment of the EDEM Discrete Element Model of the Bowl Body

#### 3.1.1. Measurement of Physical Properties of Pot Seedling

The experiment was conducted at the Key Laboratory of Modern Agricultural Equipment and Technology, Ministry of Education, Jiangsu University. The indoor environment was maintained at a temperature of (22 ± 2) °C and a relative humidity of (50 ± 5)%. After the seedlings were transported from the nursery factory to the laboratory, they were neatly arranged on seedling tray racks and left to rest in the laboratory environment for no less than 12 h to allow their temperature and humidity conditions to fully equilibrate with the testing environment. The experiment was carried out under natural daylight to minimize the effects of location factors (light gradient, temperature variation, and test bench/plot effects). The seedlings used were 128-cell tray seedlings uniformly cultivated by the nursery factory, with the variety being Shanghai bok choy (Jingguan No. 1). The seedlings were grown in accordance with the “NY/T 2119-2012 Vegetable Tray Seedling Standard” under natural light in a Venlon-type glass greenhouse. The seedling growth cycle lasted 33 days. The substrate in the bowl body was prepared by mixing peat, vermiculite, and perlite in a volume ratio of 3:1:1, and the bowl body compaction degree was 1.2. After filling and compaction, the substrate was level with the top of the seedling tray. The temperature was maintained at (22 ± 3) °C and relative humidity at (70 ± 5)%. Watering was performed daily to ensure the substrate in the bowl body maintained a moisture content of no less than 55%. Standardized seedling cultivation techniques were employed to minimize the impact of the seedlings’ characteristics on the experimental results. According to the previous research of the research group, a moisture content of 60% is more suitable for mechanical transplanting [26]. The substrate in the bowl body was thoroughly watered by the tidal infiltration method the day before the experiment to make the substrate moisture spread evenly. Ten trays of seedlings were randomly selected before the experiment, and 20 seedlings were randomly selected from each tray. The dry weight and wet weight of the 20 bowl bodies were determined by the weighing method. The average moisture content of the sample was calculated according to the soil moisture content formula to determine whether the seedlings used in the experiment met the standard of 60% moisture content.

(1) Measurement of seedling pot structural dimensions

**Figure 5:**
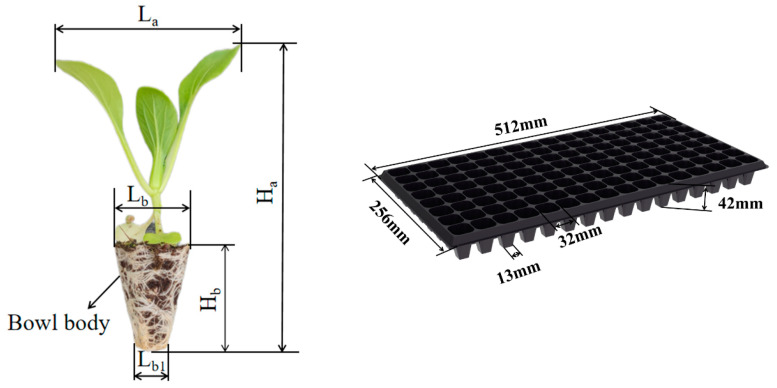
Schematic diagram of the Shanghai bok choy seedlings and 128-hole seedling tray.

During the measurement, 20 seedlings were randomly selected from the 10 selected seedling trays. The structural dimensions of the seedlings were measured using a vernier caliper (range of 200 mm, accuracy of 0.01 mm). The morphological size of the Shanghai bok choy seedlings and the seedling tray are shown in Figure 5, and the structural dimensions of the seedlings are shown in Table 2.

(2) Measurement of the density of the substrate in the bowl body

**Figure 6:**
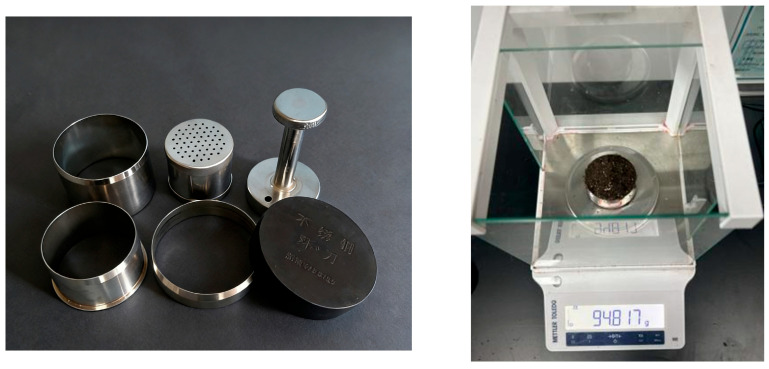
Schematic diagram of density measurement using the ring cutter method.

The density of the substrate in the seedling pots was determined using the ring cutter method. A certain volume of substrate with a moisture content of 60% was prepared, consisting of a mixture of peat moss, vermiculite, and perlite in a volume ratio of 3:1:1. A standard ring cutter (60 cm^3^) with smooth inner walls was selected. Before the test, the mass mc of the ring cutter was weighed. The ring cutter was then pressed vertically and at a uniform speed into the substrate, keeping the axis of the ring cutter perpendicular to the surface of the substrate, until the ring cutter was completely filled with substrate. Subsequently, excess substrate was scraped along the upper and lower ends of the ring cutter to level the substrate with the cutter opening, ensuring that the sampling volume was consistent with the volume of the ring cutter. The total mass mb of the ring cutter and substrate was weighed, as shown in Figure 6.

The density parameter was then calculated according to Formula (3). The average value of the ten experiments was taken as the density of the substrate in the bowl body. The experimental results are shown in Table 3. After calculation, the density of the substrate in the bowl body is 0.794 g/cm^3^.

(3) $$\rho =\frac{m_{a}}{v}=\frac{m_{b}-m_{c}}{v}$$

where ***ρ*** is the density of the substrate in the bowl, g/cm^3^; *m*_a_ is the weight of the substrate, g; *m*_b_ is the total weight of the ring cutter and substrate, g; *m*_c_ is the weight of the ring cutter, g; and *v* is the volume of the ring cutter, cm^3^.

(3) Plate compression test of the substrate in the bowl body

**Figure 7:**
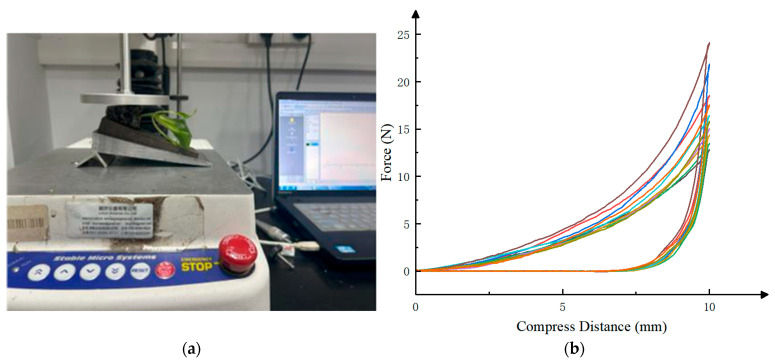
Plate compression test of the substrate in the bowl body and pressure–displacement curve of the substrate in the bowl body: (**a**) Schematic diagram of the plate compression test of the substrate in the bowl body. (**b**) Pressure–displacement curve of the substrate in the bowl body.

During the measurement, 10 seedlings were randomly selected from 10 trays. The elastic modulus E and Poisson’s ratio μ were determined using a TA.XT PlusC texture analyzer (Stable Micro Systems Ltd., Godalming, UK), as shown in Figure 7. In order to ensure that the bowl body is placed horizontally, an 11° inclined pad is needed to support the bowl body. A P/75 circular flat compression probe was employed, with a compression speed of 1 mm/s and a compression displacement of 10 mm. Data recording was initiated when the probe detected a trigger force of 0.5 N. The experimental data were recorded and processed using the texture analyzer’s experimental analysis software (Texture Lab Pro 1.1).

By repeating the compression test of the substrate in the bowl body ten times, and calculating the elastic modulus *E* and Poisson’s ratio *μ* using formulas 4 and 5, it can be concluded that the Poisson’s ratio of the potter body is 0.237 and the elastic modulus is The content in the image you mentioned will not affect scientific understanding 3.95 MPa.

(4) $$\mu =\frac{S_{h}}{S_{a}}=\frac{B_{1}-B_{2}}{C_{1}-C_{2}}$$

where *μ* is Poisson’s ratio; *S*_h_ and *S*_a_ are the horizontal and axial deformations of the substrate in the bowl body, mm; *B*_1_ and *B*_2_ are the horizontal dimensions of the substrate in the bowl body after compression, mm; and *C*_1_ and *C*_2_ are the axial dimensions of the substrate in the bowl body after compression, mm.

(5) $$E=\frac{F_{e}L}{A\cdot ∆L}$$

where *E* is the elastic modulus, MPa; *F*_e_ is the maximum force that the substrate in the bowl body can withstand during the elastic deformation stage, N; *L* is the initial length of the substrate in the bowl body, mm; and *A* is the cross-sectional area of the substrate in the bowl body, mm^2^. ∆*L* is the length difference in the substrate in the bowl body after compression.

(4) Measurement of the friction coefficient of the substrate in the bowl body

**Figure 8:**
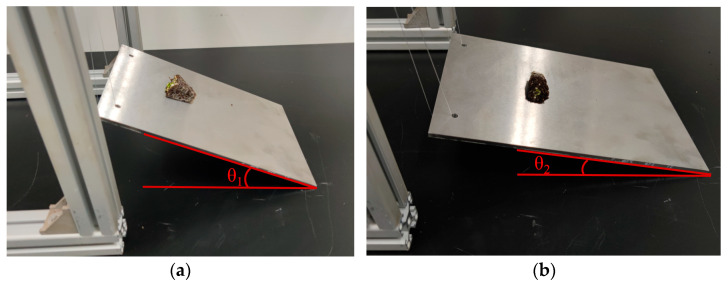
Measurement of the friction coefficient of the substrate in the bowl body: (**a**) Measurement of the static friction coefficient. (**b**) Measurement of the coefficient of rolling friction.

During the measurement, 20 seedlings were randomly selected from 10 trays. The substrate in the bowl body is placed horizontally on a steel plate made of No. 45 steel. Then, one side of the steel plate is slowly raised, and the tilt angle *θ*_1_ when the substrate in the bowl body begins to slide is recorded. The static friction coefficient is measured, as shown in Figure 8a. The substrate in the bowl body is trimmed into a cylinder with a diameter of 15 mm and placed horizontally on a steel plate. The steel plate is then slowly raised at one end. When the substrate in the bowl body begins to roll on the surface of the steel plate, the rotation is immediately stopped, and the angle of inclination *θ*_2_ between the steel plate and the table surface is recorded, as shown in Figure 8b.

The static friction coefficient between the substrate in the bowl body and the steel plate was calculated according to Formula (6), and the kinetic friction coefficient between the substrate in the bowl body and the steel plate was calculated according to Formula (7). After 10 experiments, the average value of the static friction coefficient *μ*_1_ was 0.406, and the average value of the rolling friction coefficient *μ*_2_ was 0.264.

(6) $$\mu_{1}=tan\theta_{1}$$

where *μ*_1_ is the static friction coefficient between the substrate in the bowl body and the steel plate, and *θ*_1_ is the angle between the steel plate and the horizontal plane, °.

(7) $$\mu_{2}=\frac{M}{F_{n}}=\frac{G\sin\theta_{2}}{G\cos\theta_{2}}=r\tan\theta_{2}$$

where *μ*_2_ is the rolling friction coefficient between the substrate in the bowl body and the steel plate; *θ*_2_ is the angle between the steel plate and the horizontal plane, °; *r* is the radius of the substrate in the bowl body, mm; *M* is the rolling friction torque, N⋅m; *F*_n_ is the support force exerted on the substrate in the bowl body by the inclined surface, N; and *G* is the weight of the substrate in the bowl body, N.

#### 3.1.2. Establishment of the Discrete Element Model of the Bowl Body

**Figure 9:**
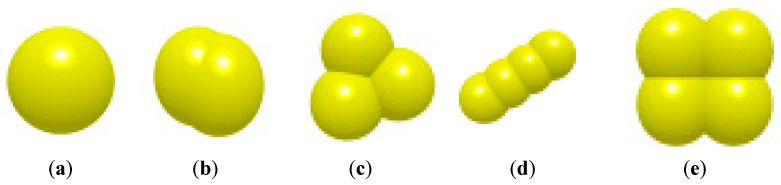
Schematic diagram of discrete element model of the substrate in the bowl body: (**a**) single-sphere; (**b**) double-sphere; (**c**) triangular; (**d**) long-strip; and (**e**) rectangular.

The substrate in the bowl body is mainly composed of peat, vermiculite, and perlite in a ratio of 3:1:1. According to the relevant literature, the basic structure of peat particles is mainly triangular particles, long-strip particles, and double spherical particles; the main structure of vermiculite is rectangular particles; and the main structure of perlite is single spherical particles [27], as shown in Figure 9. Among them, the radii of triangular particles, double-sphere particles, and long-strip particles are randomly distributed from 0.5 to 1 mm, the radii of single-sphere particles are randomly distributed from 0.1 to 0.5 mm, and the radii of rectangular particles are randomly distributed from 0.8 to 1.2 mm.

**Figure 10:**
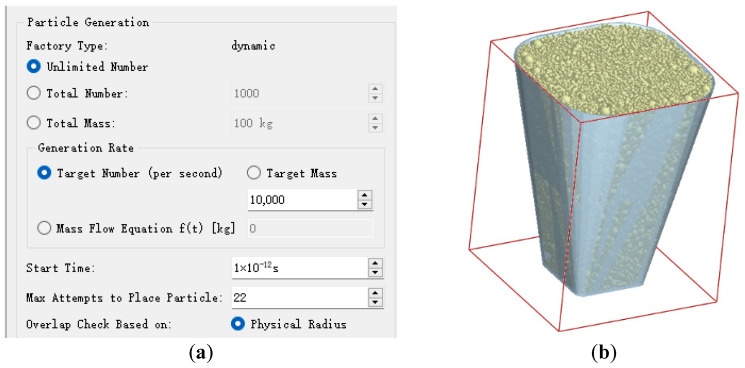
(**a**) The setup of the particle factory; (**b**) The results of particle generation.

A 1:1 model of a single hole in a 128-hole seedling tray was created using SolidWorks 2016 software. After modeling, the file was imported into EDEM2022 (FunctionBay, Inc., Troy, MI, USA) software in STP format. In EDEM2022 software, a virtual plane was created on the upper surface of the hole model, and a particle factory was created on this plane; particles were randomly and infinitely generated until the entire hole was filled. To accurately represent the contact relationship between the particles, the Hertz–Mindlin with Bonding contact model was selected. To accurately represent the contact relationship between particles and the clamping needles, and between particles and the seedling tray, the Hertz–Mindlin with JKR model was selected as the contact model [28,29]. The material of the seedling picking end-effector clamping needle is 45# steel, and the material of the seedling tray is polystyrene. The relevant material properties, contact parameters, and bonding parameters are shown in Table 4. The particle factory settings and particle generation results are shown in Figure 10.

### 3.2. Establishment of the RecurDyn VirtualPrototype Model

**Figure 11:**
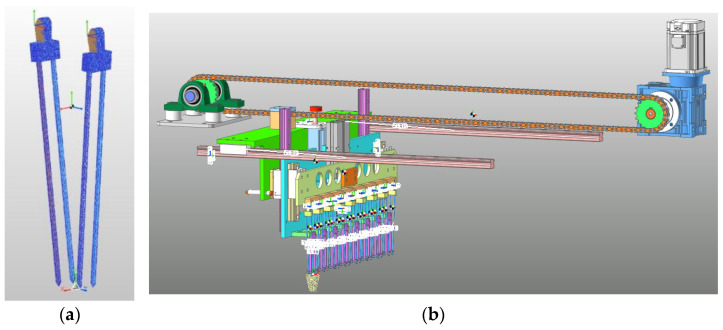
Establishment of the RecurDyn virtual prototype model: (**a**) Flexible body model of the clamping needle. (**b**) Virtual prototype model of the end effector.

Based on the structural parameters of the seedling picking end effector obtained in this study, a 3D model of the seedling picking end effector was built in SolidWorks 2016 software, and the model was imported into RecurDyn (FunctionBay, Inc., Seoul, Republic of Korea) as a Parasolid format file. Since the seedling picking end effector undergoes elastic deformation during the seedling picking process, the mesh module was used to uniformly divide the clamping needle into a flexible body, and the material was defined as No.45 steel, as shown in Figure 11a. Based on the seedling picking action of the end effector, constraints, kinematic pairs, and contact conditions were added to the entire seedling picking mechanism, as shown in Figure 11b.

### 3.3. The Setup of EDEM-RecurDyn Coupled Simulation

**Figure 12:**
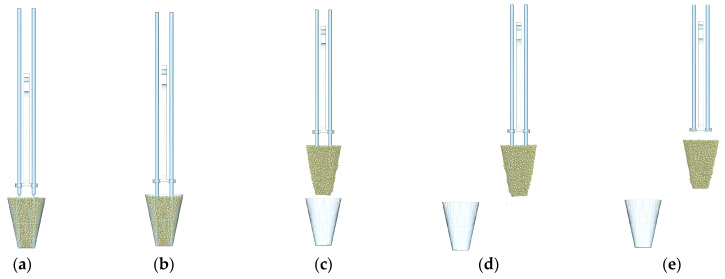
The process of EDEM-RecurDyn coupled simulation: (**a**) Preparation stage. (**b**) Inserting stage. (**c**) Extracting stage. (**d**) Transporting stage. (**e**) Throwing stage.

In the WALL module under the *External SPI* option of RecurDyn 2024, the four clamping needles and the pushing ring that contact the particles were selected to generate the WALL file, which was then imported into EDEM 2022. In EDEM 2022, the coupling server option was enabled to establish the interface for coupled simulation with RecurDyn. The simulation time and step size were configured in the *Simulator* tab of EDEM, while the corresponding parameters were set in the *Dyn*/*Kin* tab of RecurDyn 2024. The co-simulation was then initiated, and the simulation process is shown in Figure 12.

### 3.4. The Influence of the Working Parameters of the End Effector on the Particles of the Substrate in the Bowl Body

The inserting, extracting, and transporting processes of the end effector were simulated using EDEM and RecurDyn coupled simulations to examine the force, movement velocity, and number of inter-particle bonds involved in the interaction of the substrate particles. In RecurDyn, the motion expression of one working parameter was changed while the rest of the working parameters remained unchanged. The effects of different inserting and extracting velocities, transporting velocities, and inserting depths on the particles of the substrate in the bowl body were studied.

#### 3.4.1. The Influence of the Inserting and Extracting Velocity of the End Effector on the Particles of the Substrate in the Bowl Body

During the inserting and extracting of the pot seedling by the end effector, the interaction between the clamping needle and the particles of the substrate in the bowl body can cause a certain degree of disturbance to the particles, which can easily lead to substrate deformation and scattering. The inserting and extracting velocity of the end effector is the average velocity from the start of the clamping needle inserting until it is fully inserted into the bowl body, and the average speed from the start of the end effector extracting the bowl body upward until the bowl body is completely extracting. Keeping the inserting depth at 36 mm and the extracting height at 50 mm constant, coupled simulations were performed with inserting and extracting velocities of 100, 150, 200, 250, and 300 mm/s. The motion expressions are shown in Table 5. The forces and motion of the particles of the pot substrate during the inserting and extracting of the end effector were recorded.

**Figure 13:**
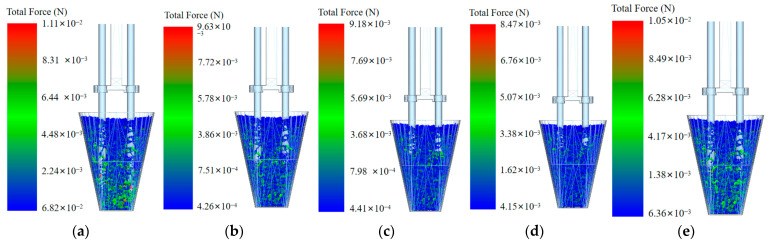
Force on particles and the number of inter-particle bonds involved in the interaction under different inserting and extracting velocities: (**a**) 100 mm/s; (**b**) 150 mm/s; (**c**) 200 mm/s; (**d**) 250 mm/s; and (**e**) 300 mm/s.

As shown in Figure 13a–d, when the inserting and extracting velocity is 100 mm/s, the pressure on the particles ranges from 6.82 × 10^−4^ to 1.11 × 10^−2^ N; when the inserting and extracting velocity is 150 mm/s, the pressure ranges from 4.26 × 10^−4^ to 9.63 × 10^−3^ N; when the inserting and extracting velocity is 200 mm/s, the pressure ranges from 4.41 × 10^−4^ to 9.18 × 10^−3^ N; and when the inserting and extracting velocity is 250 mm/s, the pressure ranges from 4.15 × 10^−4^ to 8.47 × 10^−3^ N. When the inserting and extracting velocity increases from 100 mm/s to 250 mm/s, the pressure on the particles gradually decreases, and the number of inter-particle bonds involved in the working process between particles also gradually decreases during the inserting and extracting process. As shown in Figure 12e, when the inserting and extracting velocity is 300 mm/s, the pressure exerted on the particles ranges from 6.36 × 10^−4^ to 1.05 × 10^−2^ N, and the number of inter-particle bonds involved in the interaction between particles is also relatively large. When the velocity increases to 300 mm/s, the pressure exerted on the particles and the number of inter-particle bonds involved in the interaction increase to some extent compared to the previous gradually decreasing trend. When the pressure exerted on the particles is high, the substrate particles may undergo compression or fracture, which could lead to damage to the bowl body, thereby reducing the success rate of seedling picking and throwing. The number of inter-particle bonds involved in the interaction represents the disturbance between particles caused by the inserting of the picking needles into the bowl body. The higher the number of inter-particle bonds involved in the interaction, the more particles are disturbed. As these bonds break, the particles displace, leading to the loosening or even breaking of the substrate, thus reducing the success rate of seedling picking and throwing. Therefore, the lower the pressure exerted on the particles in the bowl body, and the smaller the number of inter-particle bonds involved in the interaction, the more conducive it is to maintaining the integrity of the bowl body and improving the success rate of seedling picking and throwing.

**Figure 14:**
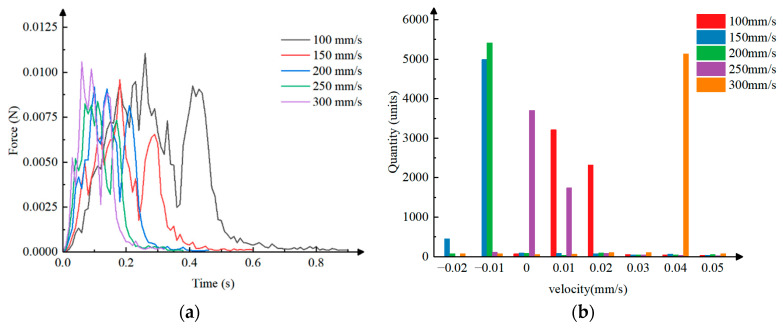
Data graphs of coupled simulation: (**a**) The pressure on the particles versus time under different inserting and extracting velocities. (**b**) The particle velocity–quantity distribution under different inserting and extracting velocities.

Data processing was performed on the coupled simulation of the inserting and extracting process, and line graphs were plotted to show the pressure on the particles versus time under different inserting and extracting velocities, as shown in Figure 14a. Analysis revealed that in the initial stage of the insertion of the clamping needle into the bowl body, the clamping needle continuously clamps the bowl body with a downward velocity, causing significant collisions with the particles. At this time, the particles need to overcome a large resistance generated by the elastic deformation of the clamping needle, resulting in significant and unstable disturbances in the bowl body, thus increasing the pressure fluctuations between the particles. As the clamping needle reaches the predetermined inserting depth and completes the clamping action, the resistance generated by the elastic deformation of the clamping needle gradually decreases. Although some disturbances still exist, the pressure between the particles shows a fluctuating decrease. When the clamping needle extracts the bowl body from the seedling tray, the particles are simultaneously subjected to pressure and friction from the clamping needle, causing a rapid increase in pressure between the particles. Once the bowl body is detached from the seedling tray, the pressure decreases rapidly. Because the machine vibrates during operation and the particles are surrounded by roots, the roots exert a force to resist the disturbance, and due to the uneven distribution of the roots, the increase and decrease in pressure are thus fluctuating. Under different inserting and extracting velocities, the pressure variation between the particles follows the same pattern as analyzed above, and the maximum pressure between the particles initially decreases and then increases with increasing inserting and extracting velocity. The lower the pressure between the particles, the better the integrity of the bowl body, and the higher the success rate of seedling picking and throwing.

The particle motion velocity along the X-axis represents the particle disturbance caused by the collision between the clamping needle and the particles. A histogram of particle velocity–quantity distribution under different inserting and extracting velocities was plotted, as shown in Figure 14b. Through analysis, it can be concluded that the velocity of the majority of the particles is between −0.02 mm/s and 0.04 mm/s. The greater the number of particles with a velocity close to 0 mm/s, the more undisturbed particles remain during the inserting and extracting process, indicating a higher degree of bowl integrity. When the inserting and extracting velocity is 100 mm/s, the particle velocity is mainly distributed at 0.01 mm/s and 0.02 mm/s, with some inter-particle disturbance. When the inserting and extracting velocity is 150 mm/s, the particle velocity is mainly distributed at −0.01 mm/s, with a small amount distributed at −0.02 mm/s, and some inter-particle disturbance exists. When the inserting and extracting velocity is 200 mm/s, the particle velocity is mostly distributed at −0.01 mm/s, with some inter-particle disturbance. When the inserting and extracting velocity is 250 mm/s, the particle velocity is mainly distributed at 0 mm/s and 0.01 mm/s, with virtually no inter-particle disturbance. When the inserting and extracting velocity is 300 mm/s, the particle velocity is mostly distributed at 0.04 mm/s, with significant inter-particle disturbance. In summary, under different inserting and extracting velocities, the inter-particle disturbance shows a trend of first decreasing and then increasing with increasing inserting and extracting velocity. The less disturbance between particles, the better the integrity of the bowl body, and the higher the success rate of seedling picking and throwing.

#### 3.4.2. The Influence of the Transporting Velocity of the End Effector on the Particles of the Substrate in the Bowl Body

The transportation process involves acceleration followed by deceleration. The clamping needle generates impact vibrations, which disturb the particles of the substrate in the bowl body, causing the substrate in the bowl body to deform and fall. The end effector’s transporting velocity is the average velocity at which the clamping needle extracts the bowl body out and transports it to the seedling throwing position. Keeping the inserting depth of 36 mm, the extracting height of 50 mm, the inserting and extracting velocity of 150 mm/s, and the transporting distance of 300 mm constant, coupled simulations were performed with transporting velocities of 250, 300, 350, 400, and 450 mm/s. The motion expressions are shown in Table 6. The forces and motion of the particles of the substrate in the bowl body during the end effector’s transporting of the pot seedling were recorded.

**Figure 15:**
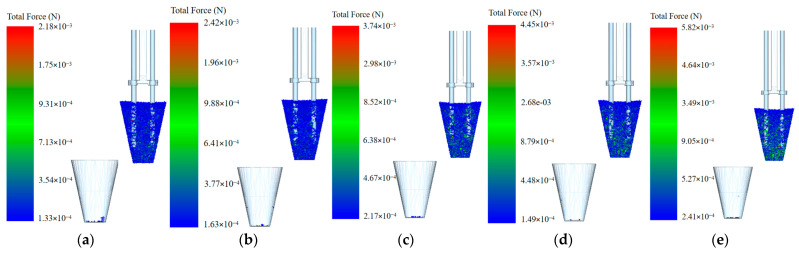
Force on particles and the number of inter-particle bonds involved in the interaction under different transporting velocities: (**a**) 250 mm/s; (**b**) 300 mm/s; (**c**) 350 mm/s; (**d**) 400 mm/s; and (**e**) 450 mm/s.

From Figure 15a–e, it can be seen that when the transporting velocity is 250 mm/s, the pressure range of the particles is 1.33 × 10^−4^~2.18 × 10^−3^ N; when the transporting velocity is 300 mm/s, the pressure range of the particles is 1.63 × 10^−4^~2.42 × 10^−3^ N; when the transporting velocity is 350 mm/s, the pressure range of the particles is 2.17 × 10^−4^~3.74 × 10^−3^ N; when the transporting velocity is 400 mm/s, the pressure range of the particles is 1.49 × 10^−4^~4.45 × 10^−3^ N; and when the transporting velocity is 450 mm/s, the pressure range of the particles is 2.41 × 10^−4^~5.82 × 10^−3^ N. When the transporting velocity increases from 250 mm/s to 450 mm/s, the pressure on the particles gradually increases, and the number of inter-particle bonds involved in the interaction between particles also gradually increases.

**Figure 16:**
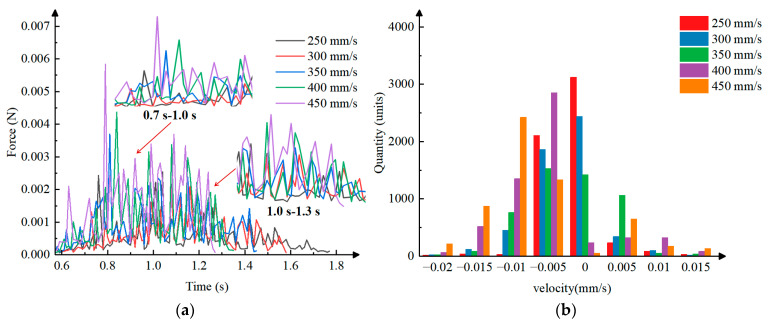
Data graphs of coupled simulation: (**a**) The pressure on the particles versus time under different transporting velocities. (**b**) The particle velocity–quantity distribution under different transporting velocities.

Data processing was performed on the coupled simulation to extract data from the transporting stage. A line graph showing the pressure on the particles versus time under different transporting velocities was plotted, as shown in Figure 16a. Analysis revealed that during the initial acceleration phase, the particle inertia is opposite to the direction of motion. At this time, the clamping needle and the particle are pressed together, and the frictional force that the clamping needle needs to overcome increases rapidly, leading to a rapid increase in pressure between the particles. During the deceleration phase, the particle inertia is in the same direction as the direction of motion, the particle spacing increases, the frictional force that the clamping needle needs to overcome decreases instantaneously, and the pressure between the particles also decreases rapidly. Because the machine vibrates during movement and the particles are surrounded by roots, the roots generate a force resisting the disturbance; therefore, the increase and decrease in pressure are fluctuating. Under different transporting velocities, the pressure variation pattern between the particles is consistent with the above analysis, and the maximum pressure between the particles gradually increases with increasing transporting velocity.

A histogram of particle velocity–quantity distribution under different transporting velocities was plotted, as shown in Figure 16b. Through analysis, it can be concluded that the velocities of the particles are mostly distributed between −0.02 mm/s and 0.015 mm/s. When the transporting velocity is 250 mm/s, the particle velocity is mainly distributed at 0 mm/s and −0.05 mm/s, and there is basically no disturbance between the particles. When the transporting velocity is 300 mm/s, the particle velocity is mainly distributed at 0 mm/s and −0.05 m/s, with a small amount distributed at −0.01 mm/s and 0.05 mm/s, and there is a small amount of disturbance between the particles. When the transporting velocity is 350 mm/s, the particle velocity is mainly distributed at 0 mm/s and ±0.05 mm/s, with a small amount distributed at −0.01 mm/s, and there is a small disturbance between the particles. When the transporting velocity is 400 mm/s, the particle velocity is mainly distributed at −0.05 mm/s, with a small part distributed at −0.01 mm/s and −0.015 mm/s, and there is a certain disturbance between the particles. When the t transporting velocity is 450 mm/s, the particle velocity is partly distributed at −0.01 mm/s, and partly distributed at ±0.05 mm/s and −0.015 mm/s, with relatively large particle disturbance. In conclusion, under different transporting velocity conditions, as the transporting velocity increases, the disturbance between particles shows a gradually increasing trend.

#### 3.4.3. The Influence of the Inserting Depths of the End Effector on the Particles of the Substrate in the Bowl Body

The inserting depth of the end effector determines the interaction area between the clamping needle and the substrate in the bowl and the degree of disturbance to the particles of the substrate in the bowl. Keeping the inserting and extracting velocity of 150 mm/s, extracting height of 50 mm, transporting distance of 300 mm, and transporting velocity of 300 mm/s constant, coupled simulations were performed with inserting depths of 35, 36, 37, 38, and 39 mm. The motion expressions are shown in Table 7. The forces and motion of the particles of the substrate in the bowl during the inserting, extracting, and transporting processes of the end effector were recorded.

**Figure 17:**
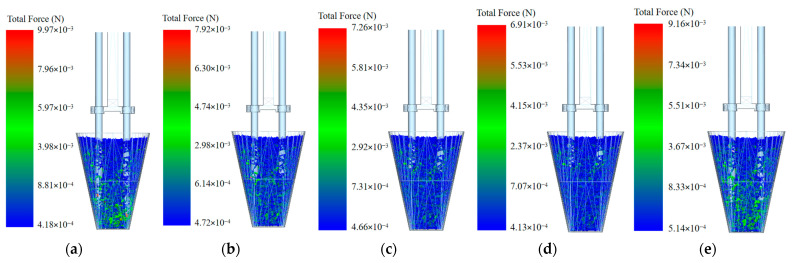
Force on particles and the number of inter-particle bonds involved in the interaction under different inserting depths: (**a**) 35 mm; (**b**) 36 mm; (**c**) 37 mm; (**d**) 38 mm; and (**e**) 39 mm.

As shown in Figure 17a–d, when the inserting depth is 35 mm, the pressure on the particles ranges from 4.18 × 10^−4^ to 9.97 × 10^−3^ N; when the inserting depth is 36 mm, the pressure ranges from 4.72 × 10^−4^ to 7.92 × 10^−3^ N; when the inserting depth is 37 mm, the pressure ranges from 4.66 × 10^−4^ to 7.26 × 10^−3^ N; and when the inserting depth is 38 mm, the pressure ranges from 4.13 × 10^−4^ to 6.91 × 10^−3^ N. When the inserting depth increases from 35 mm to 38 mm, the pressure on the particles gradually decreases. Furthermore, during inserting, extracting, and transporting, the number of bonds involved in the working process between particles also gradually decreases. As shown in Figure 17e, when the insertion depth is 38 mm, the pressure on the particles ranges from 5.14 × 10^−4^ to 9.16 × 10^−3^ N, and the number of inter-particle bonds involved in the working process between particles is also relatively large. When the inserting depth increases to 39 mm, the pressure on the particles and the number of inter-particle bonds involved in the working process increase to a certain extent compared with the previous trend of gradually decreasing.

**Figure 18:**
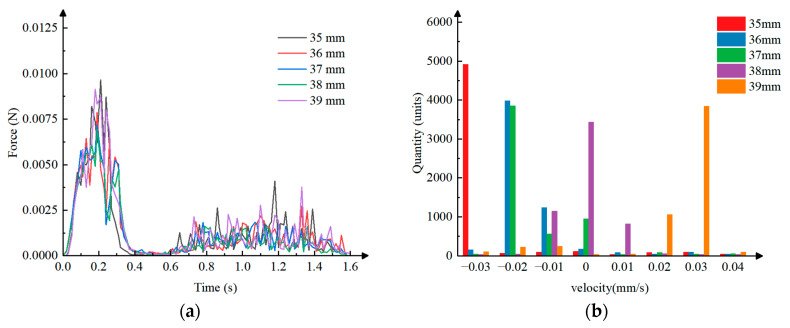
Data graphs of coupled simulation: (**a**) The pressure on the particles versus time under different inserting depths. (**b**) The particle velocity–quantity distribution under different inserting depths.

Data processing was performed on the coupled simulation of the inserting, extracting, and transporting process, and line graphs were plotted to show the pressure on the particles versus time under different inserting depths, as shown in Figure 18a. Analysis revealed that as the clamping needle continuously inserts into the bowl body, it continuously clamps the bowl body and deforms it, causing continuous disturbance to the substrate particles, leading to a rapid increase in pressure on the particles. Once the clamping needle reaches the set depth, the clamping action is complete, and there is no significant relative movement between the clamping needle and the particles, resulting in a rapid decrease in pressure. When the needle extracts the bowl body away from the seedling tray, the particles experience significant relative movement due to the friction applied by the clamping needle, causing compression between the particles and a sharp increase in pressure. When the bowl body is completely extracted, the pressure rapidly decreased and tended to stabilize. Because the machine vibrates during movement and the particles are surrounded by roots, the roots generate a force resisting the disturbance; therefore, the increase and decrease in pressure are fluctuating. Under different inserting depths, the pressure variation pattern between particles is consistent with the above analysis, and as the inserting depth increases, the maximum pressure between particles shows a trend of first decreasing and then increasing.

A histogram of particle velocity–quantity distribution under different inserting depths was plotted, as shown in Figure 18b. Through analysis, it can be concluded that the velocities of the particles are mostly distributed between −0.03 mm/s and 0.04 mm/s. When the inserting depth is 35 mm, the particle velocity is mainly distributed at −0.03 mm/s, with significant inter-particle disturbance. When the inserting depth is 35 mm, the velocity is mainly distributed at −0.02 mm/s, with a small amount at −0.01 mm/s, also with minor inter-particle disturbance. When the inserting depth is 37 mm, the velocity is mainly distributed at −0.02 mm/s, with a small amount at −0.01 mm/s and 0 mm/s, again with minor inter-particle disturbance. When the inserting depth is 38 mm, the velocity is mainly distributed at 0 mm/s, with a small portion at −0.01 mm/s and 0.01 mm/s, and virtually no inter-particle disturbance. When the inserting depth is 39 mm, the velocity is mainly distributed at 0.02 mm/s and 0.03 mm/s, with significant particle disturbance. In summary, under different inserting depth conditions, the perturbation between particles shows a trend of first decreasing and then increasing with the increase in insertion depth.

## 4. Test Optimization and Results Analysis

### 4.1. Single-Factor Test

#### 4.1.1. Test Design and Evaluation Indicators

**Figure 19:**
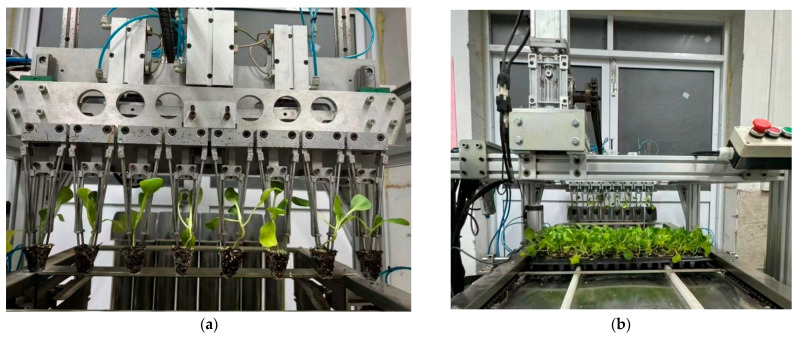
Single-factor bench: (**a**) Inserting and extracting process. (**b**) Transporting process.

The experiment was conducted at the Key Laboratory of Modern Agricultural Equipment and Technology of the Ministry of Education, Jiangsu University. The indoor environmental conditions and the experimental seedlings were consistent with the experimental conditions for measuring the physical characteristics of the seedling pots in Section 3.1.1. The success rate of seedling picking and throwing and the rate of substrate loss were used as evaluation indicators [30]. Single-factor bench tests were conducted with inserting and extracting velocity, transporting velocity, and inserting depth as single variables, the weight of the seedlings before and after seedling picking was weighed, and the number of seedlings not picked from the seedling trays, the number of seedlings that fell during transporting, and the number of seedlings with substrate weight loss exceeding 30% were recorded after the seedling picking test, as shown in Figure 19. The parameters of each factor in the test are shown in Table 8.

The formulas for the success rate of seedling picking and throwing and the loss rate of substrate loss are:

(8) $$Y_{1}=\frac{N-N_{1}-N_{2}-N_{3}}{N}\times 100\%$$

where *Y*_1_ is the success rate of seedling picking and throwing, 100%; *N* is the number of seedlings in the seedling tray, plants; *N*_1_ is the number of seedlings not picked from the seedling tray, plants; *N*_2_ is the number of seedlings that fell off during transportation, plants; and *N*_3_ is the number of seedlings with substrate weight loss exceeding 30%, plants.

(9) $$Y_{2}=\frac{M_{l}}{M_{t}}\times 100\%$$

where *Y*_2_ is the loss rate of substrate, 100%; *M*_l_ is the weight of seedlings lost during seedling picking, g; and *M*_t_ is the total weight of the entire tray of seedlings before seedling picking, g.

#### 4.1.2. Results and Analysis of the Test

**Figure 20:**
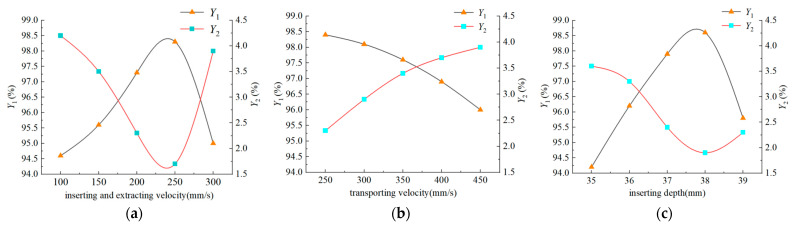
Test results: (**a**) Inserting and extracting velocity. (**b**) Transporting velocity. (**c**) Inserting depth.

A bench test was conducted on the inserting and extracting velocity, transporting velocity, and inserting depth using a single variable principle. The effects of each factor on the evaluation index are shown in Figure 20. As shown in Figure 20a, the success rate of seedling picking and throwing *Y*_1_ initially increases and then decreases with increasing inserting and extracting velocity, while the loss rate of substrate *Y*_2_ initially decreases and then increases with increasing inserting and extracting velocity. As shown in Figure 20b, the success rate of seedling picking and throwing *Y*_1_ gradually decreases with increasing transporting velocity, while the loss rate of substrate *Y*_2_ gradually increases with increasing transport speed. As shown in Figure 20c, the success rate of seedling picking and throwing *Y*_1_ initially increases and then decreases with increasing insertion depth, while the loss rate of substrate *Y*_2_ initially decreases and then slowly increases with increasing inserting depth. The results of the single-factor bench test and the coupled simulation test showed consistency. Therefore, the optimal range of the inserting and extracting velocity is 200 mm/s to 250 mm/s, the optimal range of the transporting velocity is 250 mm/s to 300 mm/s, and the optimal range of the inserting depth is 36 mm to 38 mm.

### 4.2. Multifactor Test and Optimization

#### 4.2.1. Test Design

The experiment was conducted at the Key Laboratory of Modern Agricultural Equipment and Technology of the Ministry of Education, Jiangsu University. The indoor envi-ronmental conditions and the experimental seedlings were consistent with the experi-mental conditions for measuring the physical characteristics of the seedling pots in Section 3.1.1. To investigate the influence of the above three factors on the performance of the end effector and the optimal parameter combination, a three-factor, three-level Box–Behnken bench test was designed with the success rate of seedling picking and throwing *Y*_1_ and the loss rate of substrate *Y*_2_ as evaluation indicators [31,32,33]. Based on the results of the single-factor test, the inserting and extracting velocities were set to 200, 225, and 250 mm/s, the transporting velocities were set to 250, 275, and 300 mm/s, and the inserting depths were set to 36, 37, and 38 mm. The level coding of the factors of the test is presented in Table 9.

Based on Table 9, Design-Expert 13 software was used to design tests, and a total of 17 groups of tests were designed. The design and results are shown in Table 10.

#### 4.2.2. Results and Analysis of the Test

The above results were analyzed using Design-Expert 13 software through multiple regression fitting analysis, resulting in a quadratic polynomial regression model for the success rate of seedling picking and throwing *Y*_1_ and the loss rate of substrate *Y*_2_:

(10) $$\begin{array}{c} Y_{1}=97.34-0.1500A-0.3412B-0.1912C-0.0900AB-0.1100AC-\\ 0.2625BC+0.1858A^{2}+0.0867B^{2}-0.0782C^{2} \end{array}$$

(11) $$\begin{array}{c} Y_{2}=1.95-0.5787A+0.2387B-0.1925C-0.1950AB-0.6075AC+\\ 0.2025BC-0.0900A^{2}+1.91B^{2}+0.7725C^{2} \end{array}$$

The experimental data were analyzed by ANOVA, as shown in Table 11. The results indicated that the *p*-values for the regression models of *Y*_1_ and *Y*_2_ were both <0.0001, indicating that both models are statistically significant. The lack of fit terms had *p*-values > 0.05, indicating no significant lack of fit, suggesting that the regression models fit well within the experimental range. The main effects of factors A, B, and C in both models had *p*-values < 0.01, indicating extremely significant effects on *Y*_1_ and *Y*_2_. Interaction factor BC had an extremely significant effect on *Y*_1_ (*p* < 0.01), while interaction factors AB and AC had significant effects on *Y*_1_ (*p* < 0.05). The significance of the interaction effects on *Y*_1_ ranked as follows: BC > AC > AB. Interaction factor AC had an extremely significant effect on *Y*_2_ (*p* < 0.01), while interaction factors AB and BC had significant effects on *Y*_2_ (*p* < 0.05). The significance of the interaction effects on *Y*_2_ ranked as follows: AC > BC > AB.

**Figure 21:**
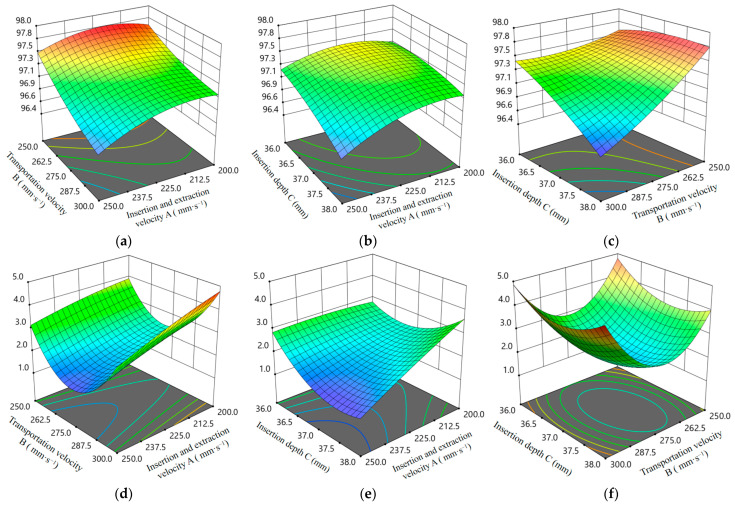
Response surface of the effects of the interaction of test factors on the success rate of seedling picking and throwing *Y*_1_ and the loss rate of substrate *Y*_2_: (**a**) The effect of AB interaction on *Y*_1_. (**b**) The effect of AC interaction on *Y*_1_. (**c**) The effect of BC interaction on *Y*_1_. (**d**) The effect of AB interaction on *Y*_2_. (**e**) The effect of AC interaction on *Y*_2_. (**f**) The effect of BC interaction on *Y*_2_.

To investigate the effects of the interaction of the above factors on the success rate of seedling picking and throwing and the loss rate of substrate, an interaction response surface was plotted [34], as shown in Figure 21. To investigate the effects of the interaction of the above factors on *Y*_1_ and *Y*_2_, an interaction response surface was plotted [34], as shown in Figure 21. Figure 21a,d: The interaction between factors A and B when C = 37 mm, and its effect on *Y*_1_ and *Y*_2_. When factor A is fixed, *Y*_1_ decreases as B increases, while *Y*_2_ decreases and then increases as B increases. When B is fixed, *Y*_1_ increases slowly and then decreases slowly as A increases, while *Y*_2_ decreases slowly as A increases. The maximum change in *Y*_1_ is 0.5%, and the maximum change in *Y*_2_ is 2.98%.

Figure 21b,e: The interaction between factors A and C when B = 275 mm/s, and its effect on *Y*_1_ and *Y*_2_. When factor A is fixed, *Y*_1_ decreases slowly as C increases. When C is fixed, *Y*_1_ increases slowly and then decreases slowly as A increases. When 200 mm/s ≤ A < 237.5 mm/s, *Y*_2_ decreases slowly and then increases as C increases; when 237.5 mm/s ≤ A < 250 mm/s, *Y*_2_ decreases first and then increases slowly as C increases. When 36 mm ≤ C < 36.5 mm, *Y*_2_ remains almost constant ·as A increases. While 36.5 mm ≤ C < 38 mm, *Y*_2_ decreases slowly as A increases. The maximum change in *Y*_1_ is 1.06%, and the maximum change in *Y*_2_ is 3.71%.

Figure 21c, f: The interaction between factors B and C when A = 225 mm/s, and its effect on *Y*_1_ and *Y*_2_. When 250 mm/s ≤ B < 275 mm/s, *Y*_1_ increases slowly with C and then remains constant; when 275 mm/s ≤ B < 300 mm/s, *Y*_1_ decreases slowly as C increases. When 36 mm ≤ C < 37 mm, *Y*_1_ decreases slowly and then remains constant as B increases; when 37 mm ≤ C < 38 mm, *Y*_1_ decreases as B increases. When B is fixed, *Y*_2_ decreases slowly and then increases slowly as C increases. When C is fixed, *Y*_2_ first decreases and then increases as B increases. The maximum change in *Y*_1_ is 1.11%, and the maximum change in *Y*_2_ is 2.87%.

In summary, under the interaction between A and B, the maximum change in *Y*_1_ is 0.5% and the maximum change in *Y*_2_ is 2.98%; under the interaction between A and C, the maximum change in *Y*_1_ is 1.06% and the maximum change in *Y*_2_ is 3.71%; under the interaction between B and C, the maximum change in *Y*_1_ is 1.11% and the maximum change in *Y*_2_ is 2.87%. Therefore, for *Y*_1_, the significance of interaction factors on its influence is as follows: BC > AC > AB, indicating that the interaction between B and C has the most significant effect on transplant success rate, with the maximum change being 1.11%. For *Y*_2_, the significance of interaction factors on its influence is as follows: AC > BC > AB, indicating that the interaction between A and C has the greatest effect on substrate loss rate, with the maximum change being 3.71%. Since *Y*_1_ and *Y*_2_ are directly related to crop growth quality and transplant cost, the changes of 1.11% and 3.71% are of high practical value in actual operations.

#### 4.2.3. Parameter Optimization and Bench Test Verification

To ensure better performance of the seedling picking end effector, with a high success rate of seedling picking and throwing and a low loss rate of substrate as optimization objectives, the working parameters of the seedling picking end effector were optimized. The optimization was solved using the Optimization-Numerical module in Design-Expert 13 software. The objective function and constraints are as follows:

(12) $$\left\{\begin{array}{l} \max Y_{1}(A,B,C)\\ \min Y_{2}(A,B,C)\\ -1\leq A\leq 1\\ -1\leq B\leq 1\\ -1\leq C\leq 1 \end{array}\right.$$

The optimal parameter combination was determined to be an inserting and extracting velocity of 228 mm/s, a transporting velocity of 264 mm/s, and an inserting depth of 37 mm, with the success rate of seedling picking and throwing of 97.48%, and the loss rate of substrate of 2.12%.

A bench test was conducted using the optimized parameters. The experiment was conducted at the Key Laboratory of Modern Agricultural Equipment and Technology of the Ministry of Education, Jiangsu University. The indoor environmental conditions and the experimental seedlings were consistent with the experimental conditions for measuring the physical characteristics of the seedling pots in Section 3.1.1. The success rate of seedling picking and throwing *Y*_1_ and the loss rate of substrate *Y*_2_ were used as evaluation indicators. Ten repeatable tests were performed, and the average value was recorded. The results are shown in Table 12. Analysis of the test data shows that the average values of the success rate of seedling picking and throwing *Y*_1_ and the loss rate of substrate *Y*_2_ were 97.35% and 2.34%, respectively, which are basically consistent with the optimized values obtained using Design-Expert 13 software. This verifies the rationality of the established model and optimized parameters.

### 4.3. Field Trial

#### 4.3.1. Trial Design and Evaluation Indicators

**Figure 22:**
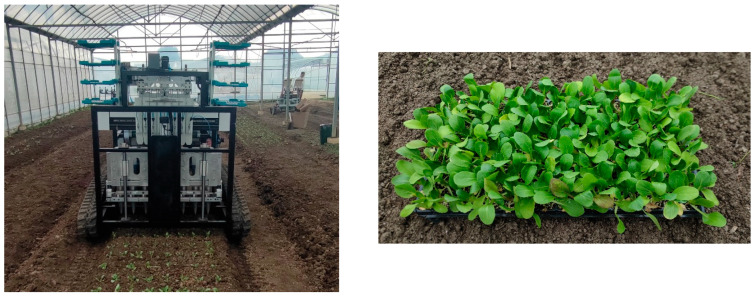
Field trial.

To test the effectiveness of the seedling picking end effector in actual operation, a field trial was conducted at the Shanghai Chunchang Vegetable and Fruit Professional Cooperative (Qingpu District, Shanghai, China). The experiment was conducted at the Key Laboratory of Modern Agricultural Equipment and Technology of the Ministry of Education, Jiangsu University. The indoor environmental conditions and the experimental seedlings were consistent with the experimental conditions for measuring the physical characteristics of the seedling pots in Section 3.1.1, as shown in Figure 22. The optimal parameter combination was selected: an inserting and extracting velocity of 228 mm/s, a transporting velocity of 264 mm/s, and an inserting depth of 37 mm. The success rate of seedling picking and throwing *Y*_1_ and the loss rate of substrate *Y*_2_ were used as evaluation indicators. Ten replicate trials were conducted, and the trial data were recorded.

#### 4.3.2. Results and Analysis of the Field Trial

The test results are shown in Table 13. Analysis of the test data shows that the success rate of seedling picking and throwing *Y*_1_ and the loss rate of substrate *Y*_2_ in the field trial were 97.04% and 2.41%, respectively. The error between the field trial success rate of seedling picking and throwing *Y*_1_ and the success rate of seedling picking and throwing *Y*_1_ optimized by Design-Expert 13 software was 0.45%. This indicates that the optimized seedling picking end effector has strong anti-interference ability when working in complex field environments, verifying the feasibility and practicality of the established model and optimized parameters.

### 4.4. Discussion

This study utilizes EDEM–RecurDyn co-simulation technology to reveal the mechanical interaction mechanisms between the seedling-picking end-effector of the automatic transplanter and the substrate in the bowl body during inserting, extracting, and transporting processes. By combining the discrete element method (EDEM) with multibody flexible dynamics (MFBD), this study provides a more accurate theoretical basis for the force and motion variations in substrate particles under complex mechanical disturbances, significantly improving the longstanding issues of unclear interactions and parameter mismatches between the end-effector and the substrate in the bowl body in previous seedling picking processes. Through the visualization analysis of pressure exerted on the particles, particle velocity, and the number of inter-particle bonds involved in the interaction, the influence mechanisms of different working parameters on the substrate particles were clarified, providing a reliable basis for optimizing the end-effector parameters. Based on the experimental results and simulation analysis, we obtained the optimal parameter combination, which increased the success rate of seedling picking and throwing to 97.48% and reduced the loss rate of substrate to 2.12%. This optimized scheme is significantly better than the traditional empirical setting, and the results of bench tests and field tests deviated from the optimized values by less than 1%, further demonstrating the effectiveness and stability of the proposed scheme in certain farmland environments.

Although our experimental tests show that these optimized parameter combinations exhibit good adaptability and stability in both bench and field tests, we speculate that, due to the limitations of the discrete element model of the bowl body in replicating the real physical properties of the bowl body, this may obscure some minor changes in the substrate during the seedling picking process. Future research could explore modeling methods for substrate particles to improve the accuracy of the model. Additionally, due to experimental limitations, especially under different varieties and climatic conditions, the success rate of seedling picking and throwing may decrease. Therefore, this speculation needs further verification through multi-season and multi-location testing, and the adaptability during long-term use still requires further study.

## 5. Conclusions

This paper addresses the problems of the low success rate of seedling picking and throwing and the high loss rate of substrate caused by unclear interaction between the end effector and the pot seedling and parameter mismatch during the seedling picking process. Coupled simulation and parameter optimization were performed on a seedling picking end effector using two fingers and four needles, and test verification was conducted. The following conclusions were drawn:

(1) Based on EDEM-Recurdyn coupled simulation, this study investigated the interaction between the end effector and substrate particles under different inserting and extracting velocities, transport velocities, and inserting depths. The results showed that with increasing velocities or inserting depth, the pressure between particles, the number of inter-particle bonds involved in the interaction, and the disturbance experienced by the particles all initially decreased and then increased. With increasing transporting velocity, the pressure between particles, the number of inter-particle bonds involved in the interaction, and the disturbance experienced by the particles all gradually increased. This resulted in lower pressure and disturbance between particles, fewer inter-particle bonds involved in the interaction, better substrate integrity, and a higher success rate of seedling picking and throwing.
(2) Single-factor bench tests were conducted, using the success rate of seedling picking and throwing and the loss rate of substrate as evaluation indicators. The test results showed consistency with the coupled simulation results. The optimal range for the inserting and extracting velocity was determined to be 200 mm/s to 250 mm/s, the optimal range for the transporting velocity was 250 mm/s to 300 mm/s, and the optimal range for the inserting depth was 36 mm to 38 mm. Three-factor, three-level Box–Behnken bench tests were conducted, and the optimal parameter combination was obtained. When the inserting and extracting velocity was 228 mm/s, the transporting velocity was 264 mm/s, the inserting depth was 37 mm, the success rate of seedling picking and throwing was 97.48%, and the loss rate of substrate was 2.12%.
(3) Bench verification tests were conducted on the optimal parameter combination. The success rate of seedling picking and throwing was 97.27%, and the loss rate of substrate was 2.36%, which were basically consistent with the optimization results, verifying the rationality of the established model and the optimized parameters. Field trials were conducted. The success rate of seedling picking and throwing was 96.98%, the loss rate of substrate was 2.42%, and the error between the success rate of seedling picking and throwing and the optimization result was 0.51%. This indicates that the established model and the optimized parameters have good reliability and engineering application value.

## Figures and Tables

**Table 1:** Geometric parameters of the seedling picking end effector.

Geometric Parameters	*L*_1_ (mm)	*L*_2_ (mm)	*L*_3_ (mm)	*L*_4_ (mm)	*H*_1_ (mm)	*H*_1′_ (mm)	*A* (°)	*α*’ (°)	*d* (mm)
Value	52	121	21	40	126	80	7	11	3

**Table 2:** Structural dimensional parameters of the Shanghai bok choy seedlings.

Parameter	Value
Height of the pot seedling *H*_a_ (mm)	110 ± 5.8
Height of the bowl body *H*_b_ (mm)	40.5 ± 0.3
Degree of leaf spread *L*_a_ (mm)	115 ± 6.2
Dimension of the upper surface of the bowl body *L*_b_ (mm)	31.5 ± 0.3
Dimension of the lower surface of the bowl body *L*_b1_ (mm)	12.6 ± 0.2

**Table 3:** Measurement results of the density of the substrate in the bowl body.

Number	*m*_c_ (g)	*m*_b_ (g)	*m*_a_ (g)	*v* (cm^3^)	*ρ* (g/cm^3^)
1	43.15	90.46	47.31	60	0.789
2	43.15	90.97	47.82	60	0.797
3	43.15	90.80	47.65	60	0.794
4	43.15	90.73	47.58	60	0.793
5	43.15	90.88	47.73	60	0.796
6	43.15	90.76	47.61	60	0.794
7	43.15	90.63	47.48	60	0.791
8	43.15	90.92	47.77	60	0.796
9	43.15	90.73	47.58	60	0.793
10	43.15	90.85	47.7	60	0.795
average					0.794

**Table 4:** Property parameters of different materials in EDEM software.

Object	Parameter	Value
Substrate particle	Poisson’s ratio	0.237
	Density (g/cm^3^)	0.794
	Elastic modulus (Mpa)	3.951
Clamping needle	Poisson’s ratio	0.269
	Density (g/cm^3^)	7.89
	Elastic modulus (Mpa)	2.0 × 10^5^
Seedling tray	Poisson’s ratio	0.35
	Density (g/cm^3^)	1.05
	Elastic modulus (Mpa)	3.375 × 10^3^
Substrate particle-Substrate particle	Recovery coefficient	0.2
	Static friction coefficient	0.647
	Kinetic friction coefficient	0.345
	Normal Stiffness per unit area (N/m^3^)	1.0 × 10^8^
	Shear Stiffness per unit area (N/m^3^)	5.0 × 10^7^
	Critical Normal Stress (Pa)	3.0 × 10^6^
	Critical Shear Stress (Pa)	1.5 × 10^6^
	Bonded Disk Radius (mm)	0.6
Substrate particle-Clamping needle	Recovery coefficient	0.6
	Static friction coefficient	0.406
	Rolling friction coefficient	0.272
	JKR surface energy (J/m^2^)	5.58
Substrate particle-Seedling tray	Recovery coefficient	0.4
	Static friction coefficient	0.441
	Rolling friction coefficient	0.272
	JKR surface energy (J/m^2^)	1.51

**Table 5:** Motion expressions under different inserting and extracting velocity conditions.

Velocity (mm/s)	Drive Components	Expression
100	Lifting cylinder	step (time, 0, 0, 0.36, −36)
	Lead screw motor	step (time, 0, 0, 0.36, 0) + step (time, 0.36, 0, 0.86, −50)
150	Lifting cylinder	step (time, 0, 0, 0.24, −36)
	Lead screw motor	step (time, 0, 0, 0.24, 0) + step (time, 0.24, 0, 0.57, −50)
200	Lifting cylinder	step (time, 0, 0, 0.18, −36)
	Lead screw motor	step (time, 0, 0, 0.18, 0) + step (time, 0.18, 0, 0.43, −50)
250	Lifting cylinder	step (time, 0, 0, 0.144, −36)
	Lead screw motor	step (time, 0, 0, 0.144, 0) + step (time, 0.144, 0, 0.344, −50)
300	Lifting cylinder	step (time, 0, 0, 0.12, −36)
	Lead screw motor	step (time, 0, 0, 0.12, 0) + step (time, 0.12, 0, 0.287, −50)

**Table 6:** Motion expressions under different transporting velocity conditions.

Velocity (mm/s)	Drive Components	Expression
250, 300, 350, 400, 450	Lifting cylinder	step(time, 0, 0, 0.24, −36)
	Lead screw motor	step(time, 0, 0, 0.24, 0) + step(time, 0.24, 0, 0.57, −50)
250	Servo motor	step(time, 0, 0, 0.57, 0) + step(time, 0.57, 0, 1.77, 300)
300		step(time, 0, 0, 0.57, 0) + step(time, 0.57, 0, 1.57, 300)
350		step(time, 0, 0, 0.57, 0) + step(time, 0.57, 0, 1.43, 300)
400		step(time, 0, 0, 0.57, 0) + step(time, 0.57, 0, 1.32, 300)
450		step(time, 0, 0, 0.57, 0) + step(time, 0.57, 0, 1.24, 300)

**Table 7:** Motion expressions under different inserting depth conditions.

Depth (mm)	Drive Components	Expression
35	Lifting cylinder	step(time, 0, 0, 0.23, −35)
	Lead screw motor	step(time, 0, 0, 0.23, 0) + step(time, 0.23, 0, 0.56, −50)
	Servo motor	step(time, 0, 0, 0.56, 0) + step(time, 0.56, 0, 1.56, 300)
36	Lifting cylinder	step(time, 0, 0, 0.24, −36)
	Lead screw motor	step(time, 0, 0, 0.24, 0) + step(time, 0.24, 0, 0.57, −50)
	Servo motor	step(time, 0, 0, 0.57, 0) + step(time, 0.57, 0, 1.57, 300)
37	Lifting cylinder	step(time, 0, 0, 0.246, 37)
	Lead screw motor	step(time,0, 0, 0.246, 0) + step(time, 0.246, 0, 0.576, −50)
	Servo motor	step(time, 0, 0, 0.576, 0) + step(time, 0.576, 0, 1.576, 300)
38	Lifting cylinder	step(time, 0, 0, 0.25, −38)
	Lead screw motor	step(time, 0, 0, 0.25, 0) + step(time, 0.25, 0, 0.58, −50)
	Servo motor	step(time, 0, 0, 0.58, 0) + step(time, 0.58, 0, 1.58, 300)
39	Lifting cylinder	step(time,0,0,0.27,−41)
	Lead screw motor	step(time, 0, 0, 0.27, 0) + step(time, 0.27, 0, 0.6, −50)
	Servo motor	step(time, 0, 0, 0.6, 0) + step(time, 0.6, 0, 1.6, 300)

**Table 8:** The parameters of each factor of the test.

Number	Factor		
	Inserting and Extracting Velocity (mm/s)	Transporting Velocity (mm/s)	Inserting Depth (mm)
1	100, 150, 200, 250, 300	300	36
2	150	300	35, 36, 37, 38, 39
3	150	250, 300, 350, 400, 450	36

**Table 9:** The level coding of the factors of the test.

Coding	Factor		
	Inserting and Extracting Velocity A (mm/s)	Transporting Velocity B (mm/s)	Inserting Depth C (mm)
−1	200	250	36
0	225	275	37
1	250	300	38

**Table 10:** The design and results of the test.

Number	Factor			*Y*_1_/%	*Y*_2_/%
	A	B	C		
1	0	0	0	97.27	1.85
2	0	0	0	97.24	2.71
3	−1	1	0	97.57	4.83
4	1	1	0	97.29	2.01
5	1	−1	0	97.33	2.81
6	−1	0	1	96.68	1.24
7	−1	0	1	97.49	4.95
8	1	0	1	96.68	4.84
9	0	0	0	97.57	3.25
10	0	−1	1	96.63	3.29
11	0	−1	−1	97.10	4.68
12	1	0	−1	97.35	2.05
13	0	1	−1	97.45	2.03
14	0	0	0	97.74	3.91
15	−1	−1	0	97.68	3.86
16	0	0	0	97.14	3.77
17	0	1	1	97.36	1.81

**Table 11:** Analysis of variance.

Source	*Y*_1_				*Y*_2_			
	Sum of Square	Degree of Freedom	Mean Square	*p*-Value	Sum of Square	Degree of Freedom	Mean Square	*p*-Value
Model	1.96	9	0.2178	<0.0001	23.82	9	2.65	<0.0001
A	0.1800	1	0.1800	0.0005	2.68	1	2.68	<0.0001
B	0.9316	1	0.9316	<0.0001	0.4560	1	0.4560	0.0019
C	0.2926	1	0.2926	0.0001	0.2965	1	0.2965	0.0060
AB	0.0324	1	0.0324	0.0356	0.1521	1	0.1521	0.0272
AC	0.0484	1	0.0484	0.0156	1.48	1	1.48	<0.0001
BC	0.2756	1	0.2756	0.0001	0.1640	1	0.1640	0.0233
A2	0.1453	1	0.1453	0.0009	0.0341	1	0.0341	0.2291
B2	0.0317	1	0.0317	0.0371	15.36	1	15.36	<0.0001
C2	0.0258	1	0.0258	0.0537	2.51	1	2.51	<0.0001
Residual	0.0336	7	0.0048		0.1375	7	0.0196	
Lack of fit	0.0137	3	0.0046	0.5081	0.0879	3	0.0293	0.2122
Pure Error	0.0199	4	0.0050		0.0496	4	0.0124	
Total	1.99	16			23.96	16		

**Table 12:** Results of the bench test.

Number	*N* (Plants)	*N*_1_ (Plants)	*N*_2_ (Plants)	*N*_3_ (Plants)	*M*_l_ (g)	*M*_t_ (g)	*Y*_1_ (%)	*Y*_2_ (%)
1	128	2	1	1	32.71	1324.37	96.88	2.47
2	128	1	1	1	30.07	1341.68	97.66	2.24
3	128	2	0	1	31.10	1329.14	97.66	2.34
4	128	1	1	2	31.72	1338.52	96.88	2.37
5	128	1	1	1	30.56	1340.26	97.66	2.28
6	128	1	2	1	30.84	1335.18	96.88	2.31
7	128	2	1	0	31.03	1331.57	97.66	2.33
8	128	1	1	1	31.22	1328.71	97.66	2.35
9	128	2	1	1	31.07	1339.24	96.88	2.32
10	128	1	0	2	31.54	1336.35	97.66	2.36
average							97.35	2.34

**Table 13:** Results of field trial.

Number	*N* (Plants)	*N*_1_ (Plants)	*N*_2_ (Plants)	*N*_3_ (Plants)	*M*_l_ (g)	*M*_t_ (g)	*Y*_1_ (%)	*Y*_2_ (%)
1	128	2	0	2	32.46	1335.68	96.88	2.43
2	128	2	1	1	30.72	1347.25	96.88	2.28
3	128	1	1	1	31.16	1325.86	97.66	2.35
4	128	0	1	2	33.55	1331.42	97.66	2.52
5	128	3	0	2	34.29	1329.23	96.10	2.58
6	128	2	0	2	32.43	1345.64	96.88	2.41
7	128	3	1	0	31.11	1335.27	96.88	2.33
8	128	1	1	2	33.03	1337.10	96.88	2.47
9	128	1	1	1	31.50	1340.51	97.66	2.35
10	128	2	1	1	31.85	1332.82	96.88	2.39
average							97.04	2.41

## Data Availability

Data are contained within the article.
